# Glucose-dependent insulinotropic peptide and risk of cardiovascular events and mortality: a prospective study

**DOI:** 10.1007/s00125-020-05093-9

**Published:** 2020-01-23

**Authors:** Amra Jujić, Naeimeh Atabaki-Pasdar, Peter M. Nilsson, Peter Almgren, Liisa Hakaste, Tiinamaija Tuomi, Lisa M. Berglund, Paul W. Franks, Jens J. Holst, Rashmi B. Prasad, Signe S. Torekov, Susana Ravassa, Javier Díez, Margaretha Persson, Olle Melander, Maria F. Gomez, Leif Groop, Emma Ahlqvist, Martin Magnusson

**Affiliations:** 1 Department of Clinical Sciences Malmö, Lund University, Clinical Research Centre, Hämtställe HS 36, Box 50332, 202 13 Malmö, Sweden; 2 Department of Cardiology, Skåne University Hospital, Inga Marie Nilssons gata 49, 20502 Malmö, Sweden; 3 Department of Clinical Sciences Malmö, Lund University, Malmö, Sweden; 4 Lund University Diabetes Centre, Lund University, Malmö, Sweden; 5 Folkhälsan Research Centre, Biomedicum, Helsinki, Finland; 6 Research Program Unit, Diabetes and Obesity, University of Helsinki, Helsinki, Finland; 7 Department of Endocrinology, Helsinki University Hospital, Helsinki, Finland; 8 Finnish Institute of Molecular Medicine, University of Helsinki, Helsinki, Finland; 9 Department of Public Health & Clinical Medicine, Umeå University, Umeå, Sweden; 10 Department of Nutrition, Harvard School of Public Health, Boston, MA USA; 11 Department of Biomedical Sciences, The Panum Institute, University of Copenhagen, Copenhagen, Denmark; 12 Novo Nordisk Foundation Center for Basic Metabolic Research, University of Copenhagen, Copenhagen, Denmark; 13 Program of Cardiovascular Diseases, CIMA, University of Navarra, Pamplona, Spain; 14 CIBERCV, Carlos III Institute of Health, Madrid, Spain; 15 Instituto de Investigación Sanitaria de Navarra (IdisNA), Pamplona, Spain; 16 Department of Cardiology and Cardiac Surgery, University of Navarra Clinic, Pamplona, Spain; 17 Department of Nephrology, University of Navarra Clinic, Pamplona, Spain; 18 Wallenberg Center for Molecular Medicine, Lund University, Malmö, Sweden

**Keywords:** Cardiovascular, Cardiovascular events, Coronary artery disease, GIP, GLP-1, Glucagon-like peptide 1, Glucose-dependent insulinotropic peptide, Mendelian randomisation, Mortality

## Abstract

**Aims/hypothesis:**

Evidence that glucose-dependent insulinotropic peptide (GIP) and/or the GIP receptor (GIPR) are involved in cardiovascular biology is emerging. We hypothesised that GIP has untoward effects on cardiovascular biology, in contrast to glucagon-like peptide 1 (GLP-1), and therefore investigated the effects of GIP and GLP-1 concentrations on cardiovascular disease (CVD) and mortality risk.

**Methods:**

GIP concentrations were successfully measured during OGTTs in two independent populations (Malmö Diet Cancer–Cardiovascular Cohort [MDC-CC] and Prevalence, Prediction and Prevention of Diabetes in Botnia [PPP-Botnia]) in a total of 8044 subjects. GLP-1 (*n* = 3625) was measured in MDC-CC. The incidence of CVD and mortality was assessed via national/regional registers or questionnaires. Further, a two-sample Mendelian randomisation (2SMR) analysis between the GIP pathway and outcomes (coronary artery disease [CAD] and myocardial infarction) was carried out using a GIP-associated genetic variant, rs1800437, as instrumental variable. An additional reverse 2SMR was performed with CAD as exposure variable and GIP as outcome variable, with the instrumental variables constructed from 114 known genetic risk variants for CAD.

**Results:**

In meta-analyses, higher fasting levels of GIP were associated with risk of higher total mortality (HR[95% CI] = 1.22 [1.11, 1.35]; *p* = 4.5 × 10^−5^) and death from CVD (HR[95% CI] 1.30 [1.11, 1.52]; *p* = 0.001). In accordance, 2SMR analysis revealed that increasing GIP concentrations were associated with CAD and myocardial infarction, and an additional reverse 2SMR revealed no significant effect of CAD on GIP levels, thus confirming a possible effect solely of GIP on CAD.

**Conclusions/interpretation:**

In two prospective, community-based studies, elevated levels of GIP were associated with greater risk of all-cause and cardiovascular mortality within 5–9 years of follow-up, whereas GLP-1 levels were not associated with excess risk. Further studies are warranted to determine the cardiovascular effects of GIP per se.

**Electronic supplementary material:**

The online version of this article (10.1007/s00125-020-05093-9) contains peer-reviewed but unedited supplementary material, which is available to authorised users.

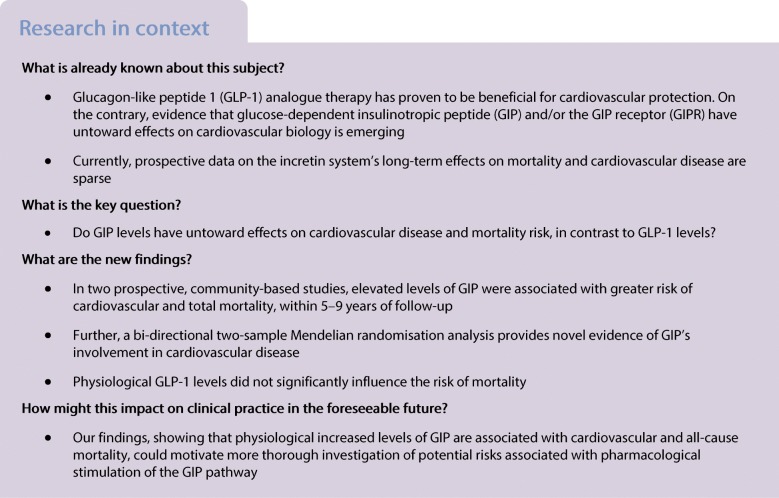



## Introduction

The enteroendocrine peptide glucose-dependent insulinotropic polypeptide (GIP) and proglucagon-derived peptides, such as glucagon-like peptide-1 (GLP-1), were classically viewed as regulators of islet function, nutrient absorption, appetite and energy homeostasis [1]. The observation that the G-protein-coupled receptors, through which these regulatory peptides exert their effects, are widely expressed in the cardiovascular system has triggered a lot of interest in their translational relevance beyond metabolic control [2].

Both experimental and clinical data, such as the outcomes from the LEADER, SUSTAIN-6, HARMONY and REWIND trials, support therapeutic benefits of GLP-1 receptor agonists with regards to cardiovascular outcomes in type 2 diabetes [3–7]. Further, a missense variant in the gene encoding the GLP-1 receptor has been associated with protection against heart disease [8]. While the bulk of the studies published so far have focused on GLP-1, GIP has received less attention. Data from our laboratory demonstrated that fasting GIP concentrations were significantly higher in individuals with a history of cardiovascular disease (CVD) than in those without, and that GIP receptor (GIPR) gene mRNA expression is higher in the arterial wall of individuals with symptoms of CVD [9]. Moreover, a common variant in *GIPR* (rs10423928), which is in complete linkage disequilibrium with rs1800437, associates with increased risk of stroke in individuals with type 2 diabetes and, recently, Ussher et al. demonstrated that reduction in GIPR signalling is linked to ischaemic cardioprotection in mice [10]. Thus, evidence that GIP and/or GIPR are involved in cardiovascular biology is emerging. In light of these findings, we explored whether circulating levels of GIP (and GLP-1) are associated with cardiovascular death and total mortality risk in two large, population-based cohorts. We also performed a two-sample Mendelian randomisation (2SMR) analysis using the *GIPR* variant rs1800437 previously associated with features of the metabolic syndrome and CVD [11] as an instrumental variable to study the effect of increased GIP levels on coronary artery disease (CAD) and myocardial infarction. Furthermore, a 2SMR analysis in a reverse direction from CAD to GIP was performed, using 114 known genetic risk variants for CAD as instrumental variables.

## Methods

### Prevalence, Prediction and Prevention of Diabetes in Botnia study

**Fig. 1:**
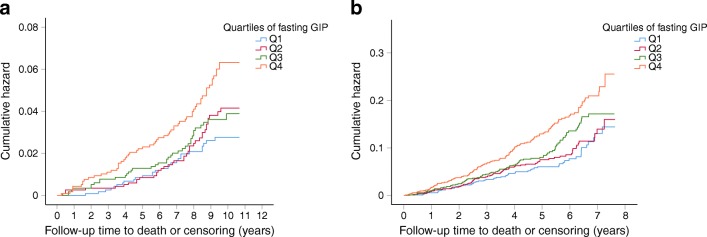
Total mortality risk in quartiles of fasting GIP. (**a**) Cumulative hazard for total mortality over a mean follow-up of 8.8 years for fasting GIP quartiles in PPP-Botnia (*p* = 0.001). (**b**) Cumulative hazard for total mortality over a mean follow-up of 5.1 years for fasting GIP quartiles (*p* = 3 × 10^−5^) in MDC-CC. Q1, quartile with the lowest values; Q4, quartile with the highest values

The Prevalence, Prediction and Prevention of Diabetes–Botnia (PPP-Botnia) study is a population-based study in western Finland started in 2004 to obtain estimates of prevalence and risk factors for type 2 diabetes, impaired glucose tolerance, impaired fasting glucose and the metabolic syndrome in the adult population. Participants were randomly recruited from the national Finnish Population Registry to represent 6–7% of the population in the 18–75 year age range (mean age 51 ± 17 years) [12]. Altogether, 5208 individuals participated in the study (54.7% of those invited). A follow-up study was conducted between 2011 and 2015, in which 3870 (74.3%) individuals participated. After exclusion of individuals with partially missing data, 4572 individuals remained for analysis of fasting GIP and 4398 for post-challenge GIP (see electronic supplementary material [ESM] Fig. 1). The number of individuals with diabetes included in analysis was 307 at the basal visit and 284 at the re-investigation visit. Diagnosis of diabetes was confirmed from participants’ records or based on fasting plasma glucose concentration ≥ 7.0 mmol/l and/or post-challenge glucose ≥ 11.1 mmol/l. The participants gave their written informed consent and the study protocol was approved by the Ethics Committee of Helsinki University Hospital, Finland.

### Malmö Diet and Cancer–Cardiovascular Cohort, Sweden

Between 1991 and 1996, a prospective, population-based study, the Malmö Diet and Cancer study, was conducted in the city of Malmö, Sweden, including questionnaires, blood sample donations and anthropometrical measurements at the baseline examination (*n* = 30,447). All people born in the years 1926–1945 and living in Malmö were invited to participate. To study cardiovascular risk factors, a sample of the study population (*n* = 6103) was randomised into a substudy, the Malmö Diet and Cancer–Cardiovascular Cohort (MDC-CC) [13]. During 2007–2012, a new clinical examination was performed (*n* = 3734) within the MDC-CC, with the addition of OGTT [14]. A schematic description of the study population is presented in ESM Fig. 2. Fasting blood samples were collected from 3692 individuals (fasting GIP available in *n* = 3479). Four-hundred-and-forty-nine individuals did not perform the complete OGTT (386 with previously known diabetes, 63 for various reasons), resulting in post-challenge (2 h) blood samples available in 3243 individuals (post-challenge GIP available in *n* = 3070). The characteristics of non-attendees at the re-examination have been described elsewhere [13]. The participants gave their written informed consent and the study protocol was approved by the Ethical Review Board, Lund, Sweden.

### Genotyping

In both cohorts, information on genotype rs1800437 was obtained from genome-wide association study data performed at the Broad genotyping facility using Illumina OmniExpressExome BeadChip v1.0 B (MDC-CC, *n* = 3344) or Illumina HumanExome BeadChip v1.0 (PPP-Botnia, *n* = 4905). The call rate was >99.9% and the SNP was in Hardy–Weinberg equilibrium in both cohorts.

### Clinical assessment

#### PPP-Botnia

Two BP recordings were obtained from the right arm of a sitting person after 30 min of rest and their mean value was calculated. If there was more than 5 mmHg difference between the two recordings, the recording was repeated. BMI was calculated as weight (kg) divided by the square of the height (m).

#### MDC-CC

BP was obtained after 10 min of rest in the supine position. BMI was calculated as weight (kg) divided by the square of the height (m).

#### OGTT

In both cohorts, a 75 g OGTT, the most appropriate method for the clinical assessment of glucometabolic status [14], was performed after an overnight fast. The OGTT was performed according to same standardised protocol in both cohorts (individuals with known diabetes did not undergo an OGTT).

### Laboratory assays

For both PPP-Botnia and MDC-CC participants, GIP was analysed by the same laboratory using the following procedure: during OGTT, blood samples were drawn in order to analyse GIP at 0 and 120 min. Serum GIP was analysed using Millipore’s Human GIP Total ELISA (Merck Millipore, Darmstadt, Germany; no. EZHGIP-54 K; minimum detection level 1.65 pmol/l, intra- and inter-assay CV 1.8–6.1% and 3–8.8%, respectively) [15].

#### PPP-Botnia

Serum insulin was measured by an AutoDelfia fluoroimmunometric assay (PerkinElmer, Waltham, MA, USA). Fasting plasma glucose was analysed using the HemoCue Glucose System (HemoCue, Ängelholm, Sweden). Serum total cholesterol, HDL-cholesterol and triacylglycerol concentrations were measured first on a Cobas Mira analyser (Hoffman-La Roche, Basel, Switzerland) and LDL-cholesterol concentrations were calculated using the Friedewald formula. Since January 2006, total cholesterol, LDL-cholesterol, HDL-cholesterol and triacylglycerol concentrations have been measured using an enzymatic method (Konelab 60i analyser; Thermo Electron Oy, Vantaa, Finland).

#### MDC-CC

During OGTT, blood samples were drawn in order to analyse GLP-1 at 0 and 120 min. Total plasma GLP-1 concentrations (intact GLP-1 and the metabolite GLP-1 9–36-amide) were determined radioimmunologically (minimum detection limit 1 pmol/l; intra- and inter-assay CV <6.0% and <15%, respectively). Identical quality controls and batches for all reagents in each analysis set were used in a consecutive sample analysis during 2 months [15]. Fasting plasma glucose was analysed using the HemoCue Glucose System. Serum insulin was assayed with Dako ELISA kit (K6219, Dako, Stockholm, Sweden; minimum detection level 3 pmol/l, intra- and inter-assay CV 5.1–7.5% and 4.2–9.3%, respectively) at the Department of Clinical Chemistry, Malmö University Hospital. HDL-cholesterol was analysed according to standard procedures at the Department of Clinical Chemistry, University Hospital Malmö. LDL-cholesterol was calculated according to the Friedewald formula.

### Classification of endpoints

#### PPP-Botnia

Mortality data were obtained from death certificates through the national registry for Causes of Death (Statistics Finland) until the end of 2014. Endpoints were defined on the basis of ICD10 codes (http://apps.who.int/classifications/icd10/browse/2016/en). Incidence and prevalence of CVD was based on a questionnaire completed by the participants at the basal and follow-up visits (for questionnaire and definitions, see ESM Methods).

Death from myocardial infarction was defined by ICD10 codes I212, I214, I219 and stroke by ICD10 codes I601, I610, I619, I620, I630, I634, I635 and I639. Death from CVD was defined by code groups I110, I119, I120, I212, I214, I219, I250, I251, I258, I259, I260, I269, I350, I420, I48, I601, I610, I619, I620, I630, I634, I635, I639, I713 and I693.

#### MDC-CC

Swedish personal identification numbers were linked to national registers (The Swedish Hospital Discharge Register and The Swedish Cause of Death Register; The National Board of Health and Welfare) [16] for cardiovascular endpoint retrieval until the end of 2014. All cardiovascular endpoints were defined on the basis of ICD8 (http://www.wolfbane.com/icd/icd8.htm), ICD9 (www.icd9data.com/2007/Volume1) and ICD10 codes. Coronary events were defined as acute myocardial infarction, other acute and subacute forms of ischaemic heart disease, old myocardial infarction, angina pectoris or other forms of chronic ischaemic heart disease, and identified using codes 410–414 (ICD8), 410–414 (ICD9) and I21, I252, I20, I251, I253-I259 (ICD10). Angioplasty events were obtained from the Swedish Coronary Angiography and Angioplasty Register (SCAAR, Kranskärlsregistret) from Uppsala Clinical Research Center, Akademiska sjukhuset, Uppsala, Sweden. Coronary artery bypass grafting was identified through original surgery codes of the first incident surgery event. The following surgery codes were extracted: Op6 (1964–96): 3065, 3066, 3068, 3080, 3092, 3105, 3127, 3158 KKÅ97 (1997–) (including subgroups). Fatal or non-fatal stroke was defined as subarachnoid haemorrhage, intracerebral haemorrhage, occlusion of cerebral arteries, acute (but ill-defined) cerebrovascular disease or stroke of unknown origin and identified using codes 430, 431, 434 and 436 (ICD9) and I60, I61, I63 and I64 (ICD10). Stroke events were extracted from Stromaregistret and Recidivregistret at the Cardiovascular epidemiology research group, SUS Malmö, Sweden. Heart failure was retrieved through codes 427.00, 427.10 and 428.99 (ICD8), 428 (ICD9) and I50 and I11.0 (ICD10). All-cause death (or otherwise emigration for censored cases) was identified through Swedish total population register Statistics Sweden, The Swedish Tax Agency and The National Board of Health and Welfare. Death from CVD was identified through codes ICD9:390–459 and ICD10:I [16–19].

### Statistical analysis

All analyses were performed in SPSS v.22.0 (SPSS, Armonk, NY, USA), except for the Mendelian randomisation (MR) analyses and the meta-analyses, which were performed using R software version 3.5.2 [20]. The 2SMR analyses were built using MendelianRandomization [21] and TwoSampleMR [22] packages. A two-tailed *p* value < 0.05 was considered significant. Skewed continuous variables were logarithmically transformed. Individuals with missing values on covariates were excluded from respective analysis.

Cox regression models were used to calculate HRs for each 1 SD increment of log-transformed fasting and post-challenge GIP and GLP-1 concentrations on mortality from CVD and total mortality risk. Individuals who died from external causes were censored.

As for analyses of incident non-fatal CVD, the two cohorts were analysed with different methods (Cox regression in MDC-CC, logistic regression in PPP-Botnia), since exact time to event was not known for PPP-Botnia. Because of this, and because endpoints were defined and recorded differently, no meta-analysis was performed. Further, in exploratory, cross-sectional analyses for associations between GIP concentration and prevalent subtypes of CVD, logistic regression was used to calculate ORs. Model 1 (adjusted for age and sex) was used for the primary analysis and further adjusted for relevant physiological covariates in Model 2 (BMI, systolic BP [SBP], fasting plasma glucose [FPG], fasting insulin, LDL-cholesterol, HDL-cholesterol and smoking for analyses of fasting GIP, and BMI, SBP, post-challenge glucose, post-challenge insulin, LDL-cholesterol, HDL-cholesterol and smoking for analyses of post-challenge GIP). Further adjustment on top of Model 2 was carried out by entering diabetes status, lipid-lowering treatment (LLT), BP-lowering treatment and educational level into Model 3. Proportional hazard assumptions were tested using Schoenfeld residuals. Fixed-effects meta-analysis of mortality variables was performed in R using the metafor package [23].

A 2SMR was performed with fasting GIP levels as exposure variable, CAD and myocardial infarction were defined as outcome variables, and rs1800437 as the instrumental variable. We applied the Wald ratio method as statistical modelling for the 2SMR analysis with a summary data for the outcomes from CARDIoGRAMplusC4D consortium and UK Biobank. In addition, to further explore the direction of association between GIP and CAD, we carried out a reverse 2SMR analysis from CAD to fasting GIP. Loci from a meta-analysis of CARDiOGRAMplusC4D and UK Biobank [24] were used for the exposure summary data and constructing the instrumental variables. The summary data for the outcome (fasting GIP) was from the MDC-CC cohort. Out of 147 SNPs in meta-analysis of CARDiOGRAMplusC4D and UK Biobank (ESM Table 1) with *p* value < 5 × 10^−8^ and *r*^2^ measure of linkage disequilibrium <0.2, 116 SNPs were selected with information also in the MDC-CC (ESM Table 2). When running MR analysis, SNPs rs472109 and rs4754698 were removed as their effect alleles were ambiguous. In total, 114 SNPs were utilised to construct the instrumental variables for 2SMR from CAD to fasting GIP. The inverse variance weighted (IVW) method, which is a widely accepted approach for 2SMR analyses with several SNPs as instrumental variables, was used for the main analysis. The sensitivity analyses for the pleiotropy effect was performed using the MR Egger method [25].

## Results

Detailed characteristics of the study populations are presented in Tables 1 and 2.

**Table 1:** Characteristics of the study population within quartiles of GIP plasma concentrations in PPP-Botnia

Characteristic	Total	Q1	Q2	Q3	Q4
Fasting GIP					
No. of participants	4572	1128	1163	1157	1124
Age, years	49.7 ± 15.8	48.0 ± 15.6	49.5 ± 15.7	50.9 ± 15.9	50.4 ± 15.7
Female sex	2421 (53.0)	650 (57.6)	607 (52.2)	615 (53.3)	549 (48.8)
BMI, kg/m^2^	26.5 ± 4.4	26.1 ± 4.5	26.4 ± 4.1	26.5 ± 4.3	27.0 ± 4.8
Fasting GIP, pmol/l	31.7 (21.7–46.1)	16.6 (13.4–19.2)	26.7 (24.2–29.3)	37.9 (34.8–41.8)	61.4 (52.8–77.1)
Fasting insulin, pmol/l	38.2 (26.1–56.5)	36.3 (25.1–52.7)	36.1 (25.8–53.1)	38.5 (25.8–57.4)	42.8 (28.3–68.3)
FPG, mmol/l	5.4 ± 0.9	5.4 ± 0.7	5.4 ± 0.8	5.4 ± 0.8	5.5 ± 1.4
SBP, mmHg	133.6 ± 19.3	131.4 ± 18.5	133.0 ± 18.7	134.6 ± 20.3	135.3 ± 19.6
LDL-cholesterol, mmol/l	3.3 (2.6–3.9)	3.2 (2.6–3.8)	3.2 (2.6–3.9)	3.3 (2.7–4.0)	3.3 (2.7–3.9)
HDL-cholesterol, mmol/l	1.37 (1.13–1.65)	1.40 (1.16–1.66)	1.40 (1.14–1.68)	1.37 (1.13–1.65)	1.33 (1.10–1.62)
Diabetes	272 (5.9)	37 (3.3)	50 (4.3)	70 (6.1)	115 (10.3)
Number of events (total mortality)	154 (3.0)	26	36	36	56
GIPR rs1800437, MAF	0.28	0.33	0.27	0.27	0.24
Post-challenge GIP					
No. of participants	4398	1034	1104	1131	1129
Age, years	49.6 ± 15.7	45.6 ± 15.1	47.0 ± 15.7	50.3 ± 15.3	55.0 ± 15.0
Female sex	2326 (52.9)	414 (40.0)	518 (46.9)	625 (55.3)	769 (68.1)
BMI, kg/m^2^	26.4 ± 4.4	26.7 ± 4.7	26.4 ± 4.4	26.3 ± 4.3	26.2 ± 4.1
Post-challenge GIP, pmol/l	178.3 (131.5–237.4)	100.1 (82.3–114.3)	153.5 (140.7–163.4)	202.9 (188.7–217.8)	294.1 (259.7–349.5)
Post-challenge insulin, pmol/l	168.8 (100.0–284.0)	126.4 (63.2–222.9)	156.9 (93.1–267.4)	183.3 (111.8–297.9)	200.0 (129.9–327.0)
Post-challenge glucose, mmol/l	5.5 ± 2.2	5.3 ± 2.5	5.5 ± 2.2	5.4 ± 1.9	5.8 ± 2.3
SBP, mmHg	133.3 ± 19.0	132.4 ± 18.5	131.5 ± 18.1	134.0 ± 19.6	135.1 ± 19.6
LDL-cholesterol, mmol/l	3.3 (2.6–3.9)	3.2 (2.5–3.7)	3.2 (2.6–3.8)	3.4 (2.8–4.0)	3.5 (2.8–4.2)
HDL-cholesterol, mmol/l	1.38 (1.14–1.66)	1.35 (1.12–1.60)	1.36 (1.13–1.64)	1.36 (1.13–1.63)	1.43 (1.18–1.73)
Diabetes	194 (4.4)	61 (5.9)	40 (3.6)	37 (3.3)	56 (5.0)
No. of events (total mortality)	130	26	24	32	48
GIPR rs1800437, MAF	0.28	0.32	0.28	0.27	0.24

Values are expressed as mean ± SD, median (25th–75th interquartile range) or *n* (%)

MAF, minor allele frequency; Q1, quartile with the lowest values; Q4, quartile with the highest values

**Table 2:** Characteristics of the study population within quartiles of GIP plasma concentrations in MDC-CC

Characteristic	Total	Q1	Q2	Q3	Q4
No. of participants	3479	871	870	867	871
Fasting GIP					
Age, years	72.4 ± 5.6	71.6 ± 5.4	72.5 ± 5.6	72.8 ± 5.6	72.9 ± 5.6
Female sex	2184 (59.1)	523 (60)	503 (57.9)	494 (57.1)	522 (59.9)
BMI, kg/m^2^	26.9 ± 4.4	26.4 ± 3.9	26.6 ± 4.0	26.8 ± 4.4	27.8 ± 5.0
Fasting GIP, pmol/l	41.2 (30.4–56.8)	24.5 (20.3–27.6)	35.9 (33.2–38.3)	47.6 (44.4–51.5)	72.9 (63.3–89.3)
Fasting insulin, pmol/l	53.5 (37.5–77.1)	47.9 (34.7–66.0)	50.0 (35.4–72.2)	54.2 (38.9–77.1)	65.3 (44.4–93.1)
FPG, mmol/l	5.9 (5.4–6.5)	5.8 (5.3–6.3)	5.8 (5.4–6.4)	5.9 (5.4–6.5)	6.1 (5.5–6.8)
SBP, mmHg	143 ± 18	141 ± 17	144 ± 19	144 ± 18	144 ± 20
LDL-cholesterol, mmol/l	3.3 (2.6–3.9)	3.4 (2.8–4.0)	3.3 (2.7–3.9)	3.2 (2.6–3.9)	3.1 (2.4–3.8)
HDL-cholesterol, mmol/l	1.4 (1.1–1.7)	1.4 (1.1–1.7)	1.4 (1.1–1.7)	1.4 (1.1–1.7)	1.3 (1.0–1.6)
Diabetes	170 (4.9)	37 (4.3)	42 (4.8)	45 (5.1)	46 (5.2)
No. of events (total mortality)	346 (10.0)	60 (6.9)	74 (8.5)	88 (10.2)	124 (14.2)
GIPR rs1800437, MAF	0.22	0.27	0.21	0.21	0.20
Post-challenge GIP					
No. of participants	3070	768	766	769	767
Age, years	72.4 ± 5.6	71.1 ± 5.6	72.0 ± 5.3	72.8 ± 5.6	73.7 ± 5.5
Female sex	1830 (59.6)	355 (46.2)	425 (55.5)	488 (63.5)	562 (73.3)
BMI, kg/m^2^	26.6 ± 4.2	27.1 ± 4.2	26.4 ± 3.9	26.5 ± 4.0	26.4 ± 4.4
Post-challenge GIP, pmol/l	222.7 (163.2–293.8)	129.7 (106.4–147.1)	193.0 (178.1–207.0)	253.7 (237.3–272.4)	356.8 (321.0–414.1)
Post-challenge insulin, pmol/l	277.0 (179.9–441.0)	257.6 (171.5–400.7)	266.7 (166.0–427.8)	273.6 (189.6–441.0)	311.1 (191.7–520.1)
Post-challenge glucose, mmol/l	6.8 (5.5–8.2)	6.6 (5.5–8.1)	6.7 (5.4–8.1)	6.8 (5.5–8.32)	7.0 (5.6–8.8)
SBP, mmHg	143 ± 19	143 ± 19	144 ± 19	143 ± 19	143 ± 19
LDL-cholesterol, mmol/l	3.3 (2.7–4.0)	3.3 (2.7–3.9)	3.4 (2.7–4.0)	3.4 (2.7–4.0)	3.3 (2.7–4.0)
HDL-cholesterol, mmol/l	1.4 (1.1–1.7)	1.4 (1.0–1.7)	1.4 (1.1–1.7)	1.4 (1.1–1.7)	1.4 (1.2–1.7)
Diabetes	165 (5.4)	47 (6.2)	38 (4.9)	34 (4.4)	46 (5.9)
No. of events (total mortality)	282 (9.2)	49 (6.4)	66 (8.6)	60 (7.8)	104 (13.6)
GIPR rs1800437, MAF	0.22	0.26	0.23	0.21	0.18

Values are expressed as mean ± SD, median (25th–75th interquartile range) or *n* (%)

MAF, minor allele frequency; Q1, quartile with the lowest values; Q4, quartile with the highest values

### Total and cardiovascular mortality

#### Fasting GIP

In Cox regression analyses, adjusted for sex and age (Model 1), each 1 SD increment of log-transformed fasting GIP concentration was associated with higher total mortality risk in both cohorts. To determine the extent to which this was mediated by known risk factors for CVD we further adjusted the analyses for BMI, FPG, fasting insulin, SBP, LDL-cholesterol, HDL-cholesterol and smoking (Model 2), and the associations remained significant (Table 3). In Model 3, diabetes status, lipid-lowering treatment, BP-lowering treatment and educational level were included on top of the covariates in Model 2 (Table 3). The cumulative incidence of total mortality for each quartile of fasting GIP is shown in Kaplan–Meier plots (Fig. 1a, b).

**Table 3:** Associations of 1 SD of log-transformed fasting GIP and post-challenge GIP with total and cardiovascular mortality risk

Variable	PPP-Botnia		MDC-CC		Meta-analysis	
	HR (95% CI)	*p* value	HR (95% CI)	*p* value	HR (95% CI)	*p* value
Fasting GIP						
Total mortality						
Model 1^a^	1.29 (1.10, 1.50)	0.001	1.27 (1.14, 1.40)	6.0 × 10^−6^	1.28 (1.17, 1.39)	4.7 × 10^−8^
Model 2^b^	1.19 (1.02, 1.40)	0.029	1.26 (1.13, 1.40)	4.9 × 10^−5^	1.24 (1.13, 1.35)	3.0 × 10^−6^
Model 3^c^	1.21 (1.03, 1.43)	0.025	1.23 (1.09, 1.37)	4.3 × 10^−4^	1.22 (1.11, 1.35)	4.5 × 10^−5^
Cardiovascular mortality						
Model 1^a^	1.42 (1.11, 1.83)	0.007	1.24 (1.04, 1.48)	0.019	1.29 (1.12, 1.50)	5.0 × 10^−4^
Model 2^b^	1.34 (1.02, 1.76)	0.033	1.23 (1.02, 1.48)	0.029	1.26 (1.08, 1.48)	0.0029
Model 3^c^	1.41 (1.07, 1.85)	0.015	1.25 (1.03, 1.51)	0.023	1.30 (1.11, 1.52)	0.0012
Post-challenge GIP						
Total mortality						
Model 1^a^	0.97 (0.81, 1.17)	0.755	1.24 (1.09, 1.40)	0.001	1.15 (1.03, 1.28)	0.01
Model 2^b^	1.00 (0.83, 1.20)	0.960	1.27 (1.11, 1.45)	0.001	1.18 (1.06, 1.32)	0.004
Model 3^c^	1.00 (0.83, 1.22)	0.978	1.23 (1.07, 1.41)	0.003	1.14 (1.02, 1.28)	0.02
Cardiovascular mortality						
Model 1^a^	1.05 (0.76, 1.44)	0.771	1.50 (1.21, 1.85)	2.4 × 10^−5^	1.32 (1.10, 1.57)	0.0023
Model 2^b^	1.10 (0.80, 1.53)	0.556	1.56 (1.24, 1.96)	1.7 × 10^−4^	1.39 (1.16, 1.63)	5.0 × 10^−4^
Model 3^c^	1.10 (0.79, 1.52)	0.578	1.51 (1.20, 1.91)	4.9 × 10^−4^	1.36 (1.13, 1.65)	0.0013

No. of individuals (events) included in analyses for fasting GIP: total mortality in PPP-Botnia *n* = 4572 (154), in MDC-CC *n* = 3472 (346) and in meta-analysis *n* = 8044 (500); cardiovascular mortality in PPP-Botnia *n* = 4571 (53), in MDC *n* = 3472 (120) and in meta-analysis *n* = 8043 (173). No. of individuals (events) included in analyses for post-challenge GIP: total mortality in PPP-Botnia *n* = 4398 (130), in MDC-CC *n* = 3060 (279) and in meta-analysis: *n* = 7458 (409); cardiovascular mortality in PPP-Botnia *n* = 4398 (46), in MDC-CC *n* = 2827 (89) and in meta-analyses *n* = 7225 (135)

^a^Model 1 is adjusted for age and sex

^b^Model 2 is adjusted for age, sex, BMI, SBP, fasting or post-challenge glucose, fasting or post-challenge insulin, LDL-cholesterol, HDL-cholesterol and smoking

^c^Model 3 is adjusted for lipid-lowering treatment, BP-lowering treatment, diabetes status and educational level on top of covariates in Model 2

Increased fasting GIP concentration was also associated with risk of cardiovascular mortality in all models (Table 3).

#### Post-challenge GIP

Increased GIP concentrations after a standard OGTT (post-challenge) were associated with higher risk of total and cardiovascular mortality in a meta-analysis of the two cohorts in all models. However, the post-challenge associations were driven mainly by the MDC-CC cohort (Table 3).

#### Sensitivity analysis

To rule out the possibility that the higher mortality risk was a result of individuals being diabetic and hence contributing to a larger extent to total mortality, analyses were carried out on associations of GIP and risk of mortality, as well as cardiovascular mortality, in both cohorts excluding individuals with prevalent diabetes. The associations between GIP and mortality risk essentially remained unchanged (ESM Results, ESM Tables 3–5).

### Incident non-fatal cardiovascular events

#### Fasting GIP

Next, we analysed associations between GIP concentrations and incident, non-fatal CVD. Fasting GIP concentration was associated with incident CVD during a mean follow-up of 8.8 years in all models in PPP-Botnia. In MDC-CC, fasting GIP was not associated with higher risk of incident non-fatal CVD (Table 4).

**Table 4:** Associations of 1 SD of log-transformed fasting GIP and post-challenge GIP with incident non-fatal cardiovascular events

Variable	PPP-Botnia		MDC-CC	
	OR (95% CI)	*p* value	HR (95% CI)	*p* value
Fasting GIP				
Model 1^a^	1.33 (1.10, 1.60)	0.003	1.13 (0.98, 1.31)	0.088
Model 2^b^	1.26 (1.04, 1.52)	0.019	1.06 (0.92, 1.23)	0.406
Model 3^c^	1.25 (1.03, 1.52)	0.024	1.07 (0.93, 1.24)	0.362
Post-challenge GIP				
Model 1^a^	1.20 (0.98, 1.47)	0.073	0.98 (0.83, 1.14)	0.758
Model 2^b^	1.24 (1.01, 1.53)	0.040	0.97 (0.82, 1.15)	0.748
Model 3^c^	1.25 (1.02, 1.54)	0.035	0.97 (0.82, 1.15)	0.708

No. of individuals and incident non-fatal cardiovascular events included in analyses: for PPP-Botnia *n* = 3084 (128) and for MDC *n* = 2708 (202). The two cohorts were analysed with different methods (logistic regression in PPP-Botnia, Cox regression in MDC-CC), since exact time to event was not known for PPP-Botnia. Because of this, and because endpoints were defined and recorded differently, no meta-analysis was performed

^a^Model 1 is adjusted for age and sex

^b^Model 2 is adjusted for age, sex, BMI, SBP, fasting or post-challenge glucose, fasting or post-challenge insulin, LDL-cholesterol, HDL-cholesterol and smoking

^c^Model 3 is adjusted for lipid-lowering treatment, BP-lowering treatment, diabetes status and educational level on top of covariates in Model 2

#### Post-challenge GIP

The post-challenge GIP concentration was associated with non-fatal incident CVD in PPP-Botnia. In MDC-CC, the associations were not significant (Table 4).

A cross-sectional, exploratory analysis of GIP concentrations and CVD prevalence is presented in ESM Results.

#### Fasting and post-challenge GLP-1

Corresponding analyses were performed for GLP-1 in 3625 subjects but no significant associations were observed for either fasting or post-challenge levels of GLP-1 and mortality risk, or CVD subgroups in the MDC-CC study (ESM Table 6). GLP-1 was not measured at the basal visit in the PPP-Botnia cohort.

### MR analyses

We performed a 2SMR analysis by Wald ratio method between GIP levels as exposure and CAD (*n* = 184,305; 60,801 cases, 123,504 controls) and myocardial infarction (*n* = 171,875; 43,676 cases, 128,199 controls) as outcome variables in CARDIoGRAMplusC4D. The same procedure was applied using data from UK Biobank (CAD: *n* = 296,525; 34,541 cases, 261,984 controls) [24]. For the exposure (GIP), the initial sample was 3344 individuals of Swedish ancestry; 4905 individuals of Finnish ancestry were used as the replication sample [26]. We utilised rs1800437 as the instrumental variable (see ESM Table 7 for more details). The results show a significant association between fasting GIP and both CAD (*p* = 0.002) and myocardial infarction (*p* = 0.013), as presented in Table 5 using CARDIoGRAMplusC4D data, and significant associations between fasting GIP and CAD using UK Biobank data (*p* = 0.001). Further, a reverse 2SMR analysis was carried out with CAD as exposure and GIP as outcome variable (Table 5; detailed analysis in ESM Table 8). The non-significant 2SMR result using the IVW method (*p* = 0.148) shows that there is no directional association from CAD to GIP. There was no evidence of pleiotropy found through the MR Egger method (Table 5; *p* = 0.595). The single SNP MR estimates using each of the 114 SNPs used in the reverse MR from CAD to GIP can be found in ESM Table 9. The bi-directional MR analysis confirmed the possible direction solely from GIP to CAD.

**Table 5:** MR analyses

Exposure	Outcome	Method	IV	β	SE	*p* value
Fasting GIP^a^	CAD	Wald ratio (2SMR)	rs1800437	0.51	0.165	0.002
Fasting GIP^a^	MI	Wald ratio (2SMR)	rs1800437	0.46	0.186	0.013
Fasting GIP^b^	CAD	Wald ratio (2SMR)	rs1800437	0.42	0.129	0.001
CAD^c^	Fasting GIP	IVW	114 SNPs	−0.042	0.029	0.148
CAD^c^	Fasting GIP	MR Egger	114 SNPs	−0.039	0.074	0.595

^a^Data from CARDIoGRAMplusC4D for CAD (*n* = 184,305; 60,801 cases, 123,504 controls) and myocardial infarction (*n* = 171,875; 43,676 cases, 128,199 controls)

^b^Data for CAD from UK Biobank (*n* = 296,525; 34,541 cases, 61,984 controls)

^c^Loci from CARDiOGRAMplusC4D and UK Biobank were used for constructing the instrumental variable. The summary data for the outcome (fasting GIP) was acquired from the MDC-CC cohort. Out of 147 SNPs in CARDiOGRAMplusC4D and UK Biobank (ESM Table 1) with *p* < 5 × 10^−8^ and *r*^2^ measure of linkage disequilibrium <0.2, 116 SNPs were selected with available information in MDC-CC (ESM Table 2). An additional two SNPs (rs472109, rs4754698) were removed from analysis for being palindromic with intermediate allele frequencies

IV, instrumental variable; MI, myocardial infarction

## Discussion

This observational study demonstrates that high plasma concentration of fasting GIP is associated with higher risk of total and cardiovascular mortality in two general populations. Further, using a 2SMR, we demonstrated an association between increased GIP levels and CAD.

The results from the two studied populations were generally comparable, with the most consistent effect being the association between fasting GIP concentration and mortality risk. The discrepancies found may be explained by the mean age difference between the two populations (72 years for MDC-CC vs 50 years for PPP-Botnia), resulting in fewer outcomes (mortality and cardiovascular mortality) for PPP-Botnia and there may be differences in the underlying pathology of CVD at different ages. Other potential reasons are differences in population and lifestyle, and the sources and definitions of CVD in the two studies (self-reported for PPP and register-derived for MDC-CC). However, the associations between increased GIP levels and higher risk of CAD/myocardial infarction were confirmed using the large CARDIoGRAMplusC4D data in 2SMR analysis.

Recently, Ussher et al. showed that genetic elimination of GIPR improved survival rate and reduced adverse cardiac remodelling following experimental myocardial infarction in mice [10]. Furthermore, epidemiological studies have shown that fasting GIP concentrations are significantly higher in individuals with a history of CVD and *GIPR* mRNA expression is higher in the arterial wall of individuals with symptoms of CVD [9]. A suggested mediator of the possible cardiovascular detrimental effects of GIP is osteopontin (OPN) [9, 27, 28]. OPN regulates synthesis of extracellular matrix and the proliferation and migration of endothelial and vascular smooth muscle cells during repair and remodelling of blood vessels. OPN also promotes inflammation and recruitment of leucocytes to the vessel wall [29]. Accordingly, plasma OPN has been associated with the presence and severity of CAD in humans [30]. Notably, GIP stimulation increases OPN expression in mouse arteries and individuals with symptomatic CVD have higher plaque expression of *GIPR* and *OPN* (also known as *SPP1*) mRNA. Further, GIP infusion increases plasma concentration of OPN in humans and this effect is strongest in carriers of the minor allele of the *GIPR* rs10423928 locus [9]. Interestingly, there is also a known CAD locus (rs46522) in the *UBE2Z* gene, suggested to be mediated by a non-synonymous coding SNP (rs2291725) in the *GIP* gene, but the effect of this locus on GIP function and expression is still poorly understood [31, 32]. While recent experimental data do not actually support a direct damaging effect of GIP on cardiac cells [10, 33], recent clinical observations in obese individuals with hyperglycaemia and insulin resistance show an association of increased circulating GIP levels with biomarkers of chronic low-grade inflammation (this, in turn, might facilitate CVD) [34].

The MR associations between GIP and CAD shown in our study indicate a direct role for GIPR signalling in the pathways leading to these endpoints, although we cannot conclude that all of the risk increase observed in this study is due to direct effects of GIP on the cardiovascular system. The risk increase for death may, as an example, be mediated by unhealthy fat distribution, independent of insulin levels, that is associated with higher GIP release, or by promotion of obesity [35, 36]. However, our analyses were adjusted for BMI, implicating other pathways. Another possibility is that the association is mediated by effects on glucose homeostasis and risk of diabetes. This was addressed by adjusting for fasting glucose and insulin values in Model 2 and addition of diabetes status in Model 3. We also did a set of analyses wherein we excluded all diabetic individuals (prevalent and incident diabetes cases) in the MDC-CC cohort (ESM Tables 3–5), with associations between GIP and mortality risk essentially unchanged. In the PPP-Botnia cohort, the diabetes status of individuals who did not attend the follow-up visit could not be determined. Instead, we analysed risk of incident and total CVD in individuals who were normoglycaemic both at baseline and at follow-up and found that all associations remained in this smaller subset (ESM Table 4). A third possibility is that part of the associations could be due to unmeasured covariates.

The LEADER, SUSTAIN-6, HARMONY and REWIND studies [3–7] found lower rates of cardiovascular events among high-risk individuals with type 2 diabetes treated with the GLP-1 analogues liraglutide, semaglutide, albiglutide and dulaglutide, respectively, vs placebo. In our study, neither fasting nor post-challenge GLP-1 concentrations were associated with the risk of CVD or death, nor did we find any protective effects of GLP-1 on mortality and CVD risk. This discrepancy could be due either to the different populations studied (e.g. the PPP-Botnia and MDC-CC are population cohorts consisting of only 5.9% and 4.4% individuals with diabetes, respectively, in contrast to the LEADER and SUSTAIN trial in which only diabetic individuals were studied) or, even more likely, to different concentrations of GLP-1 as the cardioprotective effects of GLP-1 agonists/analogues demonstrated earlier are attributed to pharmacologically induced, supraphysiological levels of GLP-1 in contrast to the normal, physiological GLP-1 levels in our study.

### Strength and limitations

The use of well-characterised, prospective cohorts with many participants and a relatively long follow-up time is a significant strength of the current study. Further, we used nationwide registers with 100% coverage and high accuracy. We could not completely exclude confounding effects of unmeasured covariates linked to GIP levels but tried to minimise confounders by adjusting for relevant risk factors. Another strength of this study is the demonstration of an effect of a functional genetic variant in GIPR on CAD/myocardial infarction using the MR approach, suggesting an involvement of the GIP signalling pathway in the pathogenesis of CAD.

We acknowledge that the MR analysis has limitations such as horizontal pleiotropy. To improve the reliability of our GIP to CAD/myocardial infarction MR analysis, we considered the possible confounding phenotypes and tested for their association with our instrumental variable rs1800437, which is associated with insulin secretion [26, 28, 37], BMI and other related phenotypes (ESM Tables 10 and 11). These phenotypes are likely to mediate at least some of the association between the genetic variant in GIP and CAD (vertical pleiotropy). Because of this, and because the genetic variant affects both the concentration of GIP and the expression and function of its receptor (horizontal pleiotropy), the MR effect size estimates should be interpreted with caution. However, using a genetic variant in *GIPR* greatly strengthens the evidence that the association is due to GIP signalling, since there are no known alternative ligands for the GIPR. For the reverse MR, there was no evidence of a pleiotropic effect based on MR Egger analyses (Table 5). Furthermore, the two cohorts (PPP-Botnia and MDC-CC) differ regarding the mean age of the participants (those in PPP-Botnia were younger), event-rate and how endpoints were collected, possibly explaining the discrepancies in the results presented. Finally, our data was collected in two Nordic regions, which limits the applicability to other populations.

## Conclusion

In two prospective, community-based studies, elevated levels of GIP were associated with greater risk of all-cause and cardiovascular mortality within 5–9 years of follow-up, whereas GLP-1 levels were not associated with excess risk. Further studies are needed to determine the cardiovascular effects of GIP per se.

## Electronic supplementary material

ESM Table 1 (XLSX 53.1 kb)

ESM (PDF 578 kb)

## Data Availability

The data that support the findings of this study are available upon request from the Steering Committee of Malmö Diet and Cancer study by contacting its chair, O. Melander (olle.melander@med.lu.se). Restrictions apply to the availability of these data, which were used under license for the current study, and so they are not publicly available due to ethical and legal restrictions related to the Swedish Biobanks in Medical Care Act (2002:297) and the Personal Data Act (1998:204). Data on myocardial infarction have been contributed by CARDIoGRAMplusC4D investigators and have been downloaded from http://www.CARDIOGRAMPLUSC4D.ORG. Data on CAD have been contributed by the CARDIoGRAMplusC4D and UK Biobank CardioMetabolic Consortium CHD working group.

## Abbreviations

CAD: Coronary artery disease
CVD: Cardiovascular disease
FPG: Fasting plasma glucose
GIP: Glucose-dependent insulinotropic polypeptide
GIPR: GIP receptor
GLP-1: Glucagon-like peptide-1
IVW: Inverse variance weighted method
MDC-CC: Malmö Diet and Cancer–Cardiovascular Cohort
MR: Mendelian randomisation
OPN: Osteopontin
PPP-Botnia: Prevalence, Prediction and Prevention of Diabetes in Botnia
SBP: Systolic BP
2SMR: Two-sample Mendelian randomisation
