# Supplementary material for: Glucose-dependent insulinotropic peptide and risk of cardiovascular events and mortality: a prospective study
Source: Diabetologia. 2020 Jan 23;63(5):1043–54. doi: 10.1007/s00125-020-05093-9 (PMC7145777; doi:10.1007/s00125-020-05093-9)

GLUCOSE-DEPENDENT INSULINOTROPIC PEPTIDE AND RISK OF CARDIOVASCULAR EVENTS AND MORTALITY: A PROSPECTIVE STUDY

ELECTRONIC SUPPLEMENTARY MATERIAL (ESM)

TABLE OF CONTENTS

ESM Methods 2

Questionnaire at the follow-up visit in PPP-Botnia 2

ESM Results 2

Sensitivity analyses 2

PPP-Botnia 2

MDC-CC 2

Exploratory analysis of cardiovascular endpoints 3

PPP-Botnia 3

MDC-CC 3

ESM Table 2. Selected SNPs for construction of the instrumental variable for the reverse 2SMR analysis 4

ESM Table 3. Sensitivity analyses excluding subjects with prevalent diabetes. 8

ESM Table 4. Sensitivity analyses in normoglycemic subjects in PPP-Botnia. 9

ESM Table 5. Sensitivity analyses excluding subjects with diabetes in MDC-CC. 10

ESM Table 6. Associations of 1 SD fasting and post-challenge GLP-1 increment and outcomes in MDC-CC 11

ESM Table 7. Complete data on rs1800437 used as instrumental variable in the 2SMR analysis from GIP to CAD/MI using CARDIoGRAMplusC4D and UK Biobank datasets 12

ESM Table 8. Complete data for the reverse 2SMR analyses 14

ESM Table 9. Single SNP reverse MR analysis from CAD to GIP. 15

ESM Table 10. Associations of rs1800437 and phenotypes in UK biobank 18

ESM Table 11. Associations of rs1800437 and phenotypes in GWAS Atlas 20

ESM Fig. 1. Schematic description of the population included in PPP-Botnia study 23

ESM Fig. 2. Schematic description of the population included in Malmö Diet and Cancer Study 24

ESM Methods

Questionnaire at the follow-up visit in PPP-Botnia

Questions:

- Myocardial infarction: Has a doctor ever diagnosed you with myocardial infarction or a thrombus in the heart?
- Angina pectoris: Has a doctor ever diagnosed you with coronary disease (coronary artery stenosis or angina pectoris)?
- Coronary bypass: Have you undergone coronary bypass surgery?
- Angioplasty: Have you undergone percutaneous coronary intervention?
- Stroke: Has a doctor ever diagnosed you with stroke?

Patients who replied no to all five questions at the basal visit but yes at the follow-up were considered incident cases. Patients who replied yes at the basal visit but no at follow-up were excluded from the analysis.

ESM Results

Sensitivity analyses

In order to rule out that the increased mortality was a result of patients being diabetic and hence contributing to a larger extent to total mortality, analyses on associations of GIP and mortality as well as cardiovascular mortality in both cohorts excluding subjects with prevalent diabetes, are presented in Supplementary **ESM Table 3-5**.

PPP-Botnia

Sensitivity analysis including only individuals who were normoglycemic both at the basal visit and at follow-up were performed. Logistic regression analysis was performed for incident CVD and total CVD (including both prevalent at baseline and at follow-up) in model 1 (adjusted for age and sex and model 2 (adjusted for age, sex, BMI, SBP, fasting/post-challenge plasma glucose, fasting/post-challenge insulin, LDL, HDL and smoking (**Table 4**). CVD includes both prevalent and incident cardiovascular disease. Number of cases was 62 for incident CVD and 130 for total prevalent CVD. The number of controls was 2151.

MDC-CC

Sensitivity analyses excluding subjects with prevalent (n=545) and incident (n=249) diabetes (n_{total}=794) were performed for associations of fasting GIP, post-challenge GIP, , and prevalent and incident CVD (**ESM Table 5**). Logistic regressions were carried out in model 1 (adjusted for age, sex and tome to follow-up) and model 2 (adjusted for age, sex, time to follow-up, BMI, SBP, fasting/(post-challenge glucose), fasting/(post-challenge) insulin, LDL, HDL and smoking).

Exploratory analysis of cardiovascular endpoints

Finally, we performed a cross-sectional, exploratory analysis of GIP concentrations and CVD in order to investigate if the GIP association was specific to any subtype of CVD.

PPP-Botnia

In PPP-Botnia, fasting GIP was associated with total prevalence of CVD (n=4375; 408 events; OR[CI(95%]=1.16[1.04-1.31]; p=0.009) in *Model 2.* This association was attenuated in *Model 3* (OR[CI95%]=1.10[0.98-1.24]; p=0.125). Post-challenge GIP was associated with total prevalence of CVD (n=4360; 358 events; OR[CI95%]=1.26[1.02-1.55]; p=0.031) in *Model 3*.

In cross-sectional, logistic regression analyses of the five subcategories of CVD defined in the questionnaire (myocardial infarction, angina pectoris, coronary artery bypass grafting, angioplasty and stroke), fasting GIP concentration was associated with myocardial infarction (OR[CI95%]=1.25[1.01-1.55]; p=0.048) in *Model 3* (non-significant associations not shown).

No analyses of GIP levels and prevalent heart failure were carried out in PPP-Botnia due to lack of data on heart failure.

MDC-CC

In MDC-CC, fasting GIP was associated with total prevalence of CVD at follow-up (n=3268; 638 events; OR[CI95%]=1.14[1.03-1.26]; p=0.009) in *Model 3.* As for post-challenge GIP, each 1 SD increment in post-challenge GIP was associated with total CVD at follow-up in *Model 2* (n=2898; 501 events; OR [CI95%]=1.18[1.00-1.25]; p=0.047), but the association was attenuated when the model was further adjusted according to *Model 3* (OR[CI95%]=1.08[0.97-1.21]; p=0.164).

Further, logistic regression analyses of subcategories of prevalent CVD revealed no significant associations of fasting and post-challenge GIP for any of the five subtypes of CVD (angioplasty, coronary events, major adverse cardiac events, coronary artery bypass grafting, and stroke) when adjusted according to *Model 3* (data not shown*).*

Additional exploratory analyses revealed that fasting GIP concentration was border-line significantly associated with prevalent heart failure (OR[CI95%]=1.42[1.00-2.00]; p=0.051) in *Model 3*.

ESM Table 2. Selected SNPs for construction of the instrumental variable for the reverse 2SMR analysis

SNP	beta.outcome	se.outcome	pval.outcome	eaf.outcome	effect_allele outcome	other_allele outcome	outcome
rsa55730499	-0.00886493	0.0294333	0.763295	0.0597085	T	C	GIP
rs55791371	0.0440409	0.0218724	0.0441579	0.0980407	C	A	GIP
rs7173743	-0.00627492	0.0128649	0.625764	0.417684	C	T	GIP
rs28451064	0.0152185	0.0189161	0.421164	0.136615	A	G	GIP
rs6728861	-0.0293762	0.0189901	0.122	0.128199	A	G	GIP
rs35158675	-0.00157326	0.0146871	0.914703	0.734008	A	G	GIP
rs7137828	-0.00883838	0.0129628	0.495406	0.524798	T	C	GIP
rs17514846	-0.00149197	0.0125677	0.905511	0.462449	A	C	GIP
rs1870634	-0.00745192	0.0135812	0.58326	0.657312	G	T	GIP
rs2107595	-0.0197169	0.0170269	0.246973	0.166667	A	G	GIP
rs6841581	-0.0108978	0.0191047	0.568436	0.128006	A	G	GIP
rs9515203	0.000189525	0.0139937	0.989195	0.284684	C	T	GIP
rs6743030	-0.0257944	0.0128651	0.0450633	0.463212	T	C	GIP
rs6982502	0.0157328	0.0126531	0.213829	0.557529	T	C	GIP
rs1412444	-0.00204114	0.0133372	0.878377	0.341472	T	C	GIP
rs3918226	0.00789545	0.0262827	0.763892	0.070408	T	C	GIP
rs9337951	0.00405385	0.0144867	0.779628	0.318294	A	G	GIP
rs185244	-0.0201812	0.0166022	0.224252	0.170618	T	C	GIP
rs72743461	0.0253015	0.0146955	0.0852368	0.235459	A	C	GIP
rs170041	-0.0161475	0.0140549	0.250704	0.28123	T	C	GIP
rs4803455	0.0182202	0.0126654	0.150385	0.505734	A	C	GIP
rs246600	0.0107431	0.0127215	0.398473	0.476538	T	C	GIP
rs7500448	-0.0112977	0.0150599	0.453209	0.229905	G	A	GIP
rs8003602	-0.00765551	0.0153995	0.61914	0.740993	C	T	GIP
rs62076439	0.0102806	0.0130386	0.430489	0.38581	T	G	GIP
rs3740390	-0.00481986	0.0200011	0.809589	0.106894	T	C	GIP
rs2519093	-0.0303402	0.0154323	0.049399	0.216098	T	C	GIP

rs789294	0.00260883	0.017667	0.882617	0.816861	G	C	GIP
rs7678555	0.00543061	0.0135105	0.68775	0.319377	C	A	GIP
rs667920	0.00927443	0.0152556	0.543282	0.781724	T	G	GIP
rs10857147	0.00159844	0.0144032	0.911642	0.308791	T	A	GIP
rs4678145	-0.0141828	0.0192362	0.461005	0.122324	C	G	GIP
rs10841443	-0.00152324	0.0144106	0.915826	0.719872	G	C	GIP
rs17091891	-0.0263767	0.020727	0.203278	0.103138	C	T	GIP
rs6909752	0.0176169	0.0133382	0.186685	0.329023	A	G	GIP
rs7251815	-0.00905785	0.0157044	0.564141	0.210702	T	G	GIP
rs112941079	0.00697332	0.0186001	0.707758	0.14283	G	A	GIP
rs11601507	0.0140772	0.0238999	0.555907	0.0762116	A	C	GIP
rs7199941	0.0211488	0.0127468	0.0972023	0.622231	G	A	GIP
rs11170820	0.0012169	0.0328931	0.970491	0.0420386	G	C	GIP
rs651821	-0.0444902	0.0275415	0.106343	0.942841	T	C	GIP
rs71566846	-0.0111665	0.0244241	0.64757	0.0684425	T	C	GIP
rs11107903	0.00777295	0.0245534	0.751592	0.0736325	A	G	GIP
rs1892094	0.00580316	0.0124175	0.640296	0.536629	T	C	GIP
rs472109	0.00182159	0.0129154	0.887849	0.606547	C	G	GIP
rs944172	0.000901346	0.013899	0.948298	0.708102	T	C	GIP
rs1317507	-0.026611	0.0140731	0.0587434	0.725034	C	A	GIP
rs6984210	-0.00721432	0.0272192	0.790995	0.0587918	G	C	GIP
rs17680741	-0.00973492	0.0135611	0.472908	0.318053	C	T	GIP
rs1050362	0.0149312	0.0131215	0.255257	0.374584	A	C	GIP
rs34991912	-0.0101069	0.0131526	0.442299	0.572902	C	T	GIP
rs11204892	-0.0198958	0.0171052	0.244874	0.836413	G	A	GIP
rs7617773	-0.0321014	0.0139753	0.0216943	0.706067	T	C	GIP
rs606452	0.0200713	0.0185664	0.279769	0.86219	C	A	GIP
rs1807214	-0.0164823	0.0242328	0.49646	0.0746649	C	A	GIP
rs9892152	0.00334208	0.0125905	0.790687	0.511065	C	T	GIP
rs2820315	0.000803153	0.0135639	0.952787	0.311219	T	C	GIP
rs4752700	-0.0113521	0.012987	0.382136	0.432852	G	A	GIP
rs12999907	-0.0331591	0.0180242	0.0659221	0.148397	G	A	GIP

rs4754698	0.00847552	0.012876	0.510438	0.428458	G	C	GIP
rs7998440	-0.00371292	0.0131355	0.777457	0.367752	A	G	GIP
rs1591805	0.000864777	0.0126842	0.945649	0.49104	G	A	GIP
rs11099493	0.0341273	0.0136445	0.0124371	0.308361	G	A	GIP
rs12500824	-0.00872832	0.013432	0.515865	0.670195	G	A	GIP
rs7145159	-0.0186382	0.0125634	0.13805	0.460414	C	T	GIP
rs4072980	0.0181507	0.0128958	0.159398	0.399926	A	G	GIP
rs11080107	-0.0227968	0.0125325	0.0690215	0.486877	C	T	GIP
rs273909	0.0363837	0.0189685	0.0552027	0.130128	G	A	GIP
rs11806316	-0.00222344	0.0128884	0.863046	0.399926	A	G	GIP
rs4613862	-0.0115513	0.0126117	0.359794	0.488181	C	A	GIP
rs12897	-0.00414305	0.0131472	0.752689	0.643544	A	G	GIP
rs885150	-0.00754238	0.0139502	0.588784	0.301642	C	T	GIP
rs11838267	-0.0166777	0.0181997	0.359553	0.13781	C	T	GIP
rs112635299	0.0146564	0.0428334	0.732248	0.0250542	T	G	GIP
rs1476098	-0.0294222	0.015576	0.059006	0.79379	C	A	GIP
rs2493298	-0.0184415	0.0180744	0.307675	0.14696	A	C	GIP
rs4918072	0.0203975	0.0143455	0.155179	0.281774	A	G	GIP
rs260020	0.00108413	0.0180312	0.95206	0.142053	T	C	GIP
rs61848342	-0.00307287	0.0134511	0.819315	0.361614	C	T	GIP
rs9964304	0.0028986	0.0142572	0.83891	0.283559	C	A	GIP
rs10237377	0.0131818	0.0131007	0.314415	0.36663	T	G	GIP
rs12149545	1.30595e-05	0.013525	0.99923	0.318739	A	G	GIP
rs10512861	0.00376962	0.0194293	0.846177	0.121176	T	G	GIP
rs3827066	-0.0151724	0.0184135	0.410024	0.132214	T	C	GIP
rs2832227	-0.0217493	0.0178471	0.223084	0.145796	G	A	GIP
rs6997340	0.00717378	0.0141432	0.612039	0.721236	C	T	GIP
rs7926712	-0.0138723	0.0141457	0.326842	0.707976	G	A	GIP
rs17080091	-0.0353172	0.024937	0.156816	0.0662186	T	C	GIP
rs17581137	-0.00858428	0.0146254	0.55729	0.250573	C	A	GIP
rs17081933	-0.00752313	0.0160823	0.639972	0.195795	A	T	GIP
rs2074164	0.00557679	0.016974	0.742522	0.169703	C	G	GIP

rs663640	0.0141116	0.0157882	0.371506	0.213505	T	C	GIP
rs6102343	-0.0106145	0.0153695	0.489863	0.210722	A	G	GIP
rs11613352	0.0244967	0.0141622	0.0837931	0.276367	T	C	GIP
rs3775058	0.00261694	0.0150304	0.861792	0.773349	T	A	GIP
rs582384	-0.0062151	0.0131725	0.637091	0.565447	A	C	GIP
rs7116641	-0.00160164	0.0137661	0.907387	0.295967	G	T	GIP
rs6700559	-0.00443824	0.0128246	0.729316	0.466973	T	C	GIP
rs7633770	-0.00331521	0.0126697	0.793601	0.432482	A	G	GIP
rs10267593	0.00952522	0.0159581	0.550631	0.200633	A	G	GIP
rs4266144	0.0200182	0.0145099	0.167817	0.285126	G	C	GIP
rs7797644	-0.0143683	0.0149011	0.33501	0.769942	C	T	GIP
rs36096196	0.00132836	0.0182795	0.942075	0.154722	T	C	GIP
rs11677932	-0.0122677	0.0136748	0.369741	0.331343	A	G	GIP
rs7696431	0.0420777	0.0128119	0.00103554	0.466737	G	T	GIP
rs11509880	-0.00596986	0.013497	0.658301	0.312326	A	G	GIP
rs76954792	-0.00802781	0.0146208	0.583006	0.255261	T	C	GIP
rs60154123	-0.00954037	0.0180167	0.596482	0.149733	T	C	GIP
rs1321309	0.00523504	0.0126288	0.678518	0.527007	A	G	GIP
rs10093110	0.0107192	0.0129279	0.407094	0.410402	A	G	GIP
rs2189839	0.00359731	0.0140635	0.798133	0.732591	G	A	GIP
rs6494488	-0.0030284	0.0173362	0.861339	0.842514	A	G	GIP
rs975722	0.00594124	0.0128444	0.643722	0.393837	G	A	GIP
rs3936511	0.00739936	0.017373	0.670206	0.165175	G	A	GIP
rs2145598	-0.0161768	0.0127119	0.203279	0.553187	A	G	GIP
rs1334894	-0.0370159	0.0208786	0.0763568	0.109453	T	C	GIP

ESM Table 3. Sensitivity analyses excluding subjects with prevalent diabetes.

	PPP-Botnia HR (CI 95%)	p	MDC-CC HR (CI 95%)	p	Meta-analysis HR (CI 95%)	p
**Fasting GIP**						
Total mortality[1]	1.24 (1.03-1.49)	0.022	1.31 (1.18-1.46)	4.1×10^{-7}	1.29 (1.18-1.42)	1.4×10^{-7}
Total mortality[2]	1.24 (1.03-1.49)	0.026	1.29 (1.16-1.45)	5×10^{-6}	1.28 (1.16-1.40)	4.2×10^{-7}
Cardiovascular mortality[1]	1.41 (1.03-1.93)	0.033	1.31 (1.09-1.56)	0.004	1.33 (1.14-1.56)	3.6×10^{-4}
Cardiovascular mortality[2]	1.48 (1.07-2.06)	0.019	1.26 (1.04-1.52)	0.020	1.31 (1.11-1.55)	1.2×10^{-3}
Cardiovascular mortality[3]						
**Post-challenge GIP**						
Total mortality[1]	0.96 (0.79-1.17)	0.7	1.22 (1.07-1.40)	0.003	1.13 (1.02-1.26)	0.025
Total mortality[2]	0.99 (0.81-1.21)	0.9	1.25 (1.09-1.43)	0.001	1.16 (1.04-1.30)	9.8×10^{-3}
Cardiovascular mortality [1]	0.93 (0.66-1.31)	0.7	1.42 (1.12-1.79)	0.003	1.24 (1.02-1.51)	0.032
Cardiovascular mortality[2]	0.98 (0.69-1.40)	0.9	1.49 (1.18-1.88)	0.001	1.31 (1.08-1.59)	6.4×10^{-3}

[1]*Model 1* is age and sex adjusted.
[2]*Model 2* is adjusted for age, sex, BMI, SBP, fasting/post-challenge plasma glucose, fasting/post-challenge insulin, LDL and HDL.
Number of subjects included in analyses and (events) for fasting GIP as follows: total mortality in MDC n=3309 (328), and in PPP n=4273 (125), cardiovascular mortality in MDC n=3312 (108), and PPP n=4273 (40).

ESM Table 4. Sensitivity analyses in normoglycemic subjects in PPP-Botnia.

	**OR (CI 95%)**	**p**
**Fasting GIP**		
Incident CVD[1]	1.43(1.10-1.85)	0.008
Incident CVD[2]	1.35(1.04-1.77)	0.027
Prevalent CVD[1]	1.42(1.17-1.73)	$4.2x10^{-4}$
Prevalent CVD[2]	1.36(1.11-1.66)	0.003
**Post-challenge GIP**		
Incident CVD[1]	1.43(1.06-1.94)	0.021
Incident CVD[2]	1.42(1.047-1.94)	0.029
Prevalent CVD[1]	1.27(1.02-1.57)	0.035
Prevalent CVD[2]	1.33(1.06-1.67)	0.015

Associations of fasting GIP and post-challenge GIP, with prevalent and incident CVD at follow-up in normoglycemic subjects in PPP-Botnia cohort. [1]*Model 1* is age and sex adjusted. [2]*Model 2* is adjusted for age, sex, BMI, SBP, fasting/post-challenge plasma glucose, fasting/post-challenge insulin, LDL, HDL and smoking. CVD=cardiovascular disease. Total CVD includes both prevalent and incident cardiovascular disease. Number of cases was 62 for incident CVD and 130 for total prevalent CVD. The number of controls was 2151.

ESM Table 5. Sensitivity analyses excluding subjects with diabetes in MDC-CC.

	**OR (CI 95%)**	**p**
**Fasting GIP**		
Incident CVD[1]	1.22(1.05-1.43)	0.010
Incident CVD[2]	1.22(1.01-1.47)	0.042
Total CVD[1]	1.27(1.14-.42)	$2.8x10^{-5}$
Total CVD[2]	1.18(1.05-1.33)	0.006
**Post-challenge GIP**		
Incident CVD[1]	1.33(1.13-1.56)	$4.6x10^{-4}$
Incident CVD[2]	1.25(1.02-1.53)	0.031
Total CVD[1]	1.24(1.11-1.39)	$2.3x10^{-4}$
Total CVD[2]	1.20(1.06-1.36)	0.004

Associations of fasting GIP and post-challenge GIP with prevalent and incident CVD at follow-up in subjects free from prevalent and incident diabetes in Malmö Diet and Cancer Cardiovascular Cohort. [1]*Model 1* is age and sex adjusted. [2]*Model 2* is adjusted for age, sex, BMI, SBP, fasting/post-challenge plasma glucose, fasting/post-challenge insulin, LDL, HDL and smoking. CVD=cardiovascular disease. Total CVD includes both prevalent and incident cardiovascular disease.

ESM Table 6. Associations of 1 SD fasting and post-challenge GLP-1 increment and outcomes in MDC-CC

	Hazard ratios (CI 95%)	p	Hazard ratios (CI 95%)	p
**MODEL 1**	**FASTING GLP-1**		**POST-CHALLENGE GLP-1**	
**Coronary events**	1.12 (0.94 – 1.35)	0.2	1.00 (0.82 – 1.24)	0.9
**Angioplasty**	1.07 (0.85 – 1.35)	0.6	0.98 (0.75 – 1.28)	0.9
**CABG**	0.93 (0.77 – 1.12)	0.5	0.90 (0.72 – 1.13)	0.4
**MACE**	1.01 (0.86 – 1.19)	0.9	0.92 (0.76 – 1.11)	0.4
**Heart failure**	1.81 (0.95 – 1.47)	0.1	1.04 (0.81 – 1.33)	0.8
**Stroke**	1.0 (0.86 – 1.18)	0.9	0.96 (0.81 – 1.15)	0.7
**Total mortality**	1.13 (0.99 – 1.29)	0.1	1.05 (0.94 – 1.18)	0.4
**Cardiovascular mortality**	1.10 (0.92 – 1.32)	0.3	1.04 (0.85 – 1.28)	0.7
**MODEL 2**	**FASTING GLP-1**		**POST-CHALLENGE GLP-1**	
**Coronary events**	1.12 (0.92 – 1.35)	0.2	1.04 (0.84 – 1.29)	0.7
**Angioplasty**	1.06 (0.83 – 1.36)	0.6	0.94 (0.71 – 1.25)	0.7
**CABG**	0.90 (0.74 – 1.10)	0.3	0.90 (0.71 – 1.14)	0.4
**MACE**	0.97 (0.82 – 1.14)	0.7	0.92 (0.76 – 1.13)	0.4
**Heart failure**	0.99 (0.81 – 1.22)	0.9	1.03 (0.79 – 1.35)	0.8
**Stroke**	1.03 (0.87 – 1.21)	0.8	0.96 (0.80 – 1.16)	0.7
**Total mortality**	1.11 (0.99 – 1.24)	0.1	1.03 (0.91 – 1.16)	0.7
**Cardiovascular mortality**	1.08 (0.90 – 1.29)	0.4	1.06 (0.86 – 1.30)	0.6

GLP-1=glucagon like peptide 1; CABG=coronary artery bypass surgery; MACE=major adverse coronary event. [1]*Model 1* is age and sex adjusted. [2]*Model 2* is adjusted for age, sex, BMI, SBP, fasting/post-challenge plasma glucose, fasting/post-challenge insulin, LDL, HDL and smoking.

ESM Table 7. Complete data on rs1800437 used as instrumental variable in the 2SMR analysis from GIP to CAD/MI using CARDIoGRAMplusC4D and UK Biobank datasets.

	**CARDIoGRAMplusC4D (CAD)**	**UK Biobank (CAD)**	**CARDIoGRAMplusC4D (MI)**
SNP	rs1800437	rs1800437	rs1800437
effect_allele.exposure	C	C	C
other_allele.exposure	G	G	G
effect_allele.outcome	C	C	C
other_allele.outcome	G	G	G
beta.exposure	-0.076	-0.076	-0.076
beta.outcome	-0.038	-0.032	-0.035
eaf.exposure	0.256	0.256	0.256
eaf.outcome	0.195	0.195	0.187
se.outcome	0.012	0.008	0.014
samplesize.outcome	184305	296525	171875
ncase.outcome	60801	34541	43676
ncontrol.outcome	123504	261984	128199
pval.outcome	0.002	0.0002	0.013
units.outcome	log odds	log odds	log odds
outcome	CAD	CAD	MI
consortium.outcome	CARDIoGRAMplusC4D	UK Biobank	CARDIoGRAMplusC4D
year.outcome	2015	2018	2015
pmid.outcome	26343387	29212778	26343387
category.outcome	Disease	Disease	Disease
subcategory.outcome	Cardiovascular	Cardiovascular	Cardiovascular
originalname.outcome	Coronary heart disease	Coronary heart disease	Myocardial infarction

gene.exposure	GIPR	GIPR	GIPR
se.exposure	0.010	0.010	0.010
pval.exposure	4,00E-15	4,00E-15	4,00E-15
units.exposure	unit decrease	unit decrease	unit decrease
exposure	Fasting GIP	Fasting GIP	Fasting GIP
Initial_sample_discription for rs1800347	3,344 Swedish ancestry individuals	3,344 Swedish ancestry individuals	3,344 Swedish ancestry individuals
Replication_smple_discription for rs1800347	4,905 Finnish ancestry individuals	4,905 Finnish ancestry individuals	4,905 Finnish ancestry individuals

ESM Table 8. Complete data for the reverse 2SMR analyses

Method	B	SE	p-value
**MR Egger**	-0.039	0.074	0.595
**Weighted median**	-0.028	0.051	0.583
**IVW**	-0.042	0.029	0.148
**Simple mode**	-0.026	0.106	0.804

Complete data for reverse 2SMR analysis. 114 SNP were analyzed with GIP as outcome and CAD as exposure.

ESM Table 9. Single SNP reverse MR analysis from CAD to GIP.

exposure	outcome	SNP	b	se	p
CAD	GIP	rs10093110	-0.335	0.404	0.407
CAD	GIP	rs10237377	-0.388	0.385	0.314
CAD	GIP	rs10267593	-0.265	0.443	0.551
CAD	GIP	rs1050362	0.427	0.375	0.255
CAD	GIP	rs10512861	-0.088	0.452	0.846
CAD	GIP	rs10841443	-0.033	0.313	0.916
CAD	GIP	rs10857147	0.033	0.294	0.912
CAD	GIP	rs11080107	-0.633	0.348	0.069
CAD	GIP	rs11099493	-0.875	0.350	0.012
CAD	GIP	rs11107903	-0.104	0.327	0.752
CAD	GIP	rs11170820	0.015	0.396	0.970
CAD	GIP	rs11204892	-0.406	0.349	0.245
CAD	GIP	rs112635299	-0.108	0.315	0.732
CAD	GIP	rs112941079	-0.112	0.300	0.708
CAD	GIP	rs11509880	-0.181	0.409	0.658
CAD	GIP	rs11601507	0.181	0.306	0.556
CAD	GIP	rs11613352	-0.681	0.393	0.084
CAD	GIP	rs11677932	0.361	0.402	0.370
CAD	GIP	rs11806316	0.065	0.379	0.863
CAD	GIP	rs11838267	0.327	0.357	0.359
CAD	GIP	rs12149545	-0.001	0.366	0.999
CAD	GIP	rs12500824	0.257	0.395	0.516
CAD	GIP	rs12897	0.115	0.365	0.753
CAD	GIP	rs12999907	0.691	0.376	0.066
CAD	GIP	rs1317507	0.665	0.352	0.059
CAD	GIP	rs1321309	0.187	0.451	0.678
CAD	GIP	rs1334894	0.771	0.435	0.076
CAD	GIP	rs1412444	-0.036	0.238	0.878
CAD	GIP	rs1476098	0.669	0.354	0.059
CAD	GIP	rs1591805	-0.023	0.343	0.946
CAD	GIP	rs170041	0.344	0.299	0.251
CAD	GIP	rs17080091	0.654	0.462	0.157
CAD	GIP	rs17081933	-0.188	0.402	0.640
CAD	GIP	rs17091891	0.447	0.351	0.203
CAD	GIP	rs17514846	-0.027	0.224	0.906
CAD	GIP	rs17581137	0.232	0.395	0.557
CAD	GIP	rs17680741	0.232	0.323	0.473
CAD	GIP	rs1807214	0.258	0.379	0.496
CAD	GIP	rs185244	-0.315	0.259	0.224

CAD	GIP	rs1870634	-0.124	0.226	0.583
CAD	GIP	rs1892094	-0.161	0.345	0.640
CAD	GIP	rs2074164	0.133	0.404	0.742
CAD	GIP	rs2107595	-0.263	0.227	0.247
CAD	GIP	rs2145598	0.578	0.454	0.203
CAD	GIP	rs2189839	-0.103	0.402	0.798
CAD	GIP	rs246600	0.250	0.296	0.398
CAD	GIP	rs2493298	-0.362	0.354	0.308
CAD	GIP	rs2519093	-0.552	0.281	0.049
CAD	GIP	rs260020	0.021	0.347	0.952
CAD	GIP	rs273909	0.743	0.387	0.055
CAD	GIP	rs2820315	0.022	0.377	0.953
CAD	GIP	rs2832227	-0.558	0.458	0.223
CAD	GIP	rs28451064	0.141	0.175	0.421
CAD	GIP	rs34991912	0.266	0.346	0.442
CAD	GIP	rs35158675	-0.022	0.210	0.915
CAD	GIP	rs36096196	0.028	0.389	0.942
CAD	GIP	rs3740390	0.073	0.303	0.810
CAD	GIP	rs3775058	-0.067	0.385	0.862
CAD	GIP	rs3827066	-0.361	0.438	0.410
CAD	GIP	rs3918226	0.074	0.246	0.764
CAD	GIP	rs3936511	0.200	0.470	0.670
CAD	GIP	rs4072980	-0.550	0.391	0.159
CAD	GIP	rs4266144	0.572	0.415	0.168
CAD	GIP	rs4613862	0.361	0.394	0.360
CAD	GIP	rs4678145	-0.221	0.301	0.461
CAD	GIP	rs4752700	-0.344	0.394	0.382
CAD	GIP	rs4803455	-0.380	0.264	0.150
CAD	GIP	rs4918072	0.523	0.368	0.155
CAD	GIP	rs55730499	-0.028	0.094	0.763
CAD	GIP	rs55791371	-0.380	0.189	0.044
CAD	GIP	rs582384	-0.188	0.399	0.637
CAD	GIP	rs60154123	-0.217	0.409	0.596
CAD	GIP	rs606452	0.427	0.395	0.280
CAD	GIP	rs6102343	-0.287	0.415	0.490
CAD	GIP	rs61848342	-0.085	0.374	0.819
CAD	GIP	rs62076439	0.214	0.272	0.430
CAD	GIP	rs6494488	-0.080	0.456	0.861
CAD	GIP	rs651821	0.645	0.399	0.106
CAD	GIP	rs663640	0.371	0.415	0.371
CAD	GIP	rs667920	0.189	0.311	0.543
CAD	GIP	rs6700559	0.159	0.458	0.729
CAD	GIP	rs6728861	-0.280	0.181	0.122
CAD	GIP	rs6743030	-0.453	0.226	0.045
CAD	GIP	rs6841581	-0.145	0.255	0.568

CAD	GIP	rs6909752	0.400	0.303	0.187
CAD	GIP	rs6982502	-0.315	0.253	0.214
CAD	GIP	rs6984210	-0.093	0.349	0.791
CAD	GIP	rs6997340	-0.217	0.429	0.612
CAD	GIP	rs7116641	-0.052	0.444	0.907
CAD	GIP	rs7137828	0.138	0.203	0.495
CAD	GIP	rs7145159	-0.582	0.393	0.138
CAD	GIP	rs71566846	-0.135	0.294	0.648
CAD	GIP	rs7173743	0.098	0.201	0.626
CAD	GIP	rs7199941	-0.572	0.345	0.097
CAD	GIP	rs7251815	-0.178	0.308	0.564
CAD	GIP	rs72743461	-0.436	0.253	0.085
CAD	GIP	rs7500448	0.202	0.269	0.453
CAD	GIP	rs7617773	-0.892	0.388	0.022
CAD	GIP	rs7633770	-0.111	0.422	0.794
CAD	GIP	rs7678555	0.111	0.276	0.688
CAD	GIP	rs76954792	-0.206	0.375	0.583
CAD	GIP	rs7696431	-1.357	0.413	0.001
CAD	GIP	rs7797644	-0.368	0.382	0.335
CAD	GIP	rs789294	0.041	0.276	0.883
CAD	GIP	rs7926712	-0.396	0.404	0.327
CAD	GIP	rs7998440	0.098	0.346	0.777
CAD	GIP	rs8003602	-0.142	0.285	0.619
CAD	GIP	rs885150	-0.210	0.388	0.589
CAD	GIP	rs9337951	0.075	0.268	0.780
CAD	GIP	rs944172	-0.023	0.347	0.948
CAD	GIP	rs9515203	-0.003	0.233	0.989
CAD	GIP	rs975722	0.212	0.459	0.644
CAD	GIP	rs9892152	0.101	0.382	0.791
CAD	GIP	rs9964304	0.076	0.375	0.839
CAD	GIP	All - IVW	-0.042	0.029	0.148
CAD	GIP	All - MR Egger	-0.039	0.074	0.595

ESM Table 10. Associations of rs1800437 and phenotypes in UK biobank

UKB-a:283	Arm fat mass (right)	Neale Lab	M & F	331226	Neale Lab	C	G	-0,0299312	2,1241E-23	0,00300278
UKB-a:249	Weight	Neale Lab	M & F	336227	Neale Lab	C	G	-0,0266963	2,1396E-23	0,00267845
UKB-b:16446	Basal metabolic rate	MRC-IEU	M & F	454874	MRC-IEU	G	C	0,0161212	1,6E-22	0,001651
UKB-a:291	Trunk fat mass	Neale Lab	M & F	331093	Neale Lab	C	G	-0,0297282	5,7716E-22	0,00308569
UKB-a:382	Waist circumference	Neale Lab	M & F	336639	Neale Lab	C	G	-0,0259408	1,2431E-21	0,00271488
UKB-a:264	Body fat percentage	Neale Lab	M & F	331117	Neale Lab	C	G	-0,0224267	1,3811E-21	0,00234979
UKB-b:9093	Arm predicted mass (left)	MRC-IEU	M & F	454655	MRC-IEU	G	C	0,0149536	4,1E-21	0,00158584
UKB-b:19925	Arm fat-free mass (left)	MRC-IEU	M & F	454672	MRC-IEU	G	C	0,0149178	7,2E-21	0,00159207
UKB-b:16099	Leg fat-free mass (left)	MRC-IEU	M & F	454805	MRC-IEU	G	C	0,0152882	1,6E-20	0,00164597
UKB-b:17271	Leg predicted mass (left)	MRC-IEU	M & F	454799	MRC-IEU	G	C	0,0151009	2,6E-20	0,00163526
UKB-a:286	Arm fat percentage (left)	Neale Lab	M & F	331198	Neale Lab	C	G	-0,0211072	9,2237E-20	0,0023199
UKB-a:282	Arm fat percentage (right)	Neale Lab	M & F	331249	Neale Lab	C	G	-0,0210604	1,6283E-19	0,00233064
UKB-b:19520	Arm fat-free mass (right)	MRC-IEU	M & F	454753	MRC-IEU	G	C	0,0140994	1,7E-19	0,0015615
UKB-b:16698	Arm predicted mass (right)	MRC-IEU	M & F	454746	MRC-IEU	G	C	0,0139659	2,9E-19	0,00155624
UKB-a:290	Trunk fat percentage	Neale Lab	M & F	331113	Neale Lab	C	G	-0,0243474	5,4963E-18	0,00281698
UKB-b:14310	Leg predicted mass (right)	MRC-IEU	M & F	454834	MRC-IEU	G	C	0,0140832	6,9E-18	0,00163444
UKB-b:12828	Leg fat-free mass (right)	MRC-IEU	M & F	454835	MRC-IEU	G	C	0,0141451	8,1E-18	0,00164503
UKB-b:14540	Whole body water mass	MRC-IEU	M & F	454888	MRC-IEU	G	C	0,0133147	2,5E-17	0,00157247
UKB-b:19921	Impedance of whole body	MRC-IEU	M & F	454840	MRC-IEU	G	C	-0,0161046	3,8E-17	0,00191302
UKB-b:13354	Whole body fat-free mass	MRC-IEU	M & F	454850	MRC-IEU	G	C	0,0131307	6,1E-17	0,00156989
UKB-b:7859	Impedance of arm (right)	MRC-IEU	M & F	454826	MRC-IEU	G	C	-0,0144108	4,8E-16	0,00177561
UKB-b:19379	Impedance of arm (left)	MRC-IEU	M & F	454850	MRC-IEU	G	C	-0,014142	2,1E-15	0,00178212
UKB-a:388	Hip circumference	Neale Lab	M & F	336601	Neale Lab	C	G	-0,0240713	2,2065E-15	0,00303565
UKB-a:268	Basal metabolic rate	Neale Lab	M & F	331307	Neale Lab	C	G	-0,0149389	1,9724E-13	0,00203223

UKB-a:335	Sodium in urine	Neale Lab	M & F	326831	Neale Lab	C	G	-0,0216244	4,4178E-13	0,00298581
UKB-a:289	Arm predicted mass (left)	Neale Lab	M & F	331146	Neale Lab	C	G	-0,0139458	6,7995E-13	0,00194131
UKB-b:14068	Impedance of leg (left)	MRC-IEU	M & F	454857	MRC-IEU	G	C	-0,0163908	9,3E-13	0,00229525
UKB-a:285	Arm predicted mass (right)	Neale Lab	M & F	331216	Neale Lab	C	G	-0,0135412	1,1153E-12	0,00190299
UKB-a:288	Arm fat-free mass (left)	Neale Lab	M & F	331159	Neale Lab	C	G	-0,0138316	1,2664E-12	0,00194861
UKB-b:17409	Trunk fat-free mass	MRC-IEU	M & F	454508	MRC-IEU	G	C	0,0110144	1,9E-12	0,00156358
UKB-b:9685	Trunk predicted mass	MRC-IEU	M & F	454463	MRC-IEU	G	C	0,0109187	2,5E-12	0,00155854
UKB-a:284	Arm fat-free mass (right)	Neale Lab	M & F	331221	Neale Lab	C	G	-0,0132444	4,0682E-12	0,00190978
UKB-b:7376	Impedance of leg (right)	MRC-IEU	M & F	454863	MRC-IEU	G	C	-0,0156036	7,1E-12	0,0022759
UKB-a:280	Leg fat-free mass (left)	Neale Lab	M & F	331258	Neale Lab	C	G	-0,0137622	9,1785E-12	0,00201819
UKB-a:281	Leg predicted mass (left)	Neale Lab	M & F	331253	Neale Lab	C	G	-0,0134415	2,0074E-11	0,00200449
UKB-b:4650	Comparative body size at age 10	MRC-IEU	M & F	454718	MRC-IEU	G	C	0,0117368	3,1E-11	0,00176749
UKB-a:277	Leg predicted mass (right)	Neale Lab	M & F	331285	Neale Lab	C	G	-0,0126461	2,8028E-10	0,00200429
UKB-a:276	Leg fat-free mass (right)	Neale Lab	M & F	331285	Neale Lab	C	G	-0,0126706	3,3778E-10	0,00201744
UKB-a:267	Whole body water mass	Neale Lab	M & F	331315	Neale Lab	C	G	-0,0120419	5,516E-10	0,00194104
UKB-a:266	Whole body fat-free mass	Neale Lab	M & F	331291	Neale Lab	C	G	-0,0117673	1,2693E-09	0,00193815
UKB-a:269	Impedance of whole body	Neale Lab	M & F	331284	Neale Lab	C	G	0,014152	1,4038E-09	0,00233716
UKB-a:273	Impedance of arm (left)	Neale Lab	M & F	331292	Neale Lab	C	G	0,0130194	1,8022E-09	0,00216454
UKB-a:333	Creatinine (enzymatic) in urine	Neale Lab	M & F	327525	Neale Lab	C	G	-0,017439	1,9183E-09	0,0029042
UKB-a:272	Impedance of arm (right)	Neale Lab	M & F	331279	Neale Lab	C	G	0,0128347	2,61E-09	0,00215543
UKB-a:34	Comparative body size at age 10	Neale Lab	M & F	331693	Neale Lab	C	G	-0,0121372	7,3506E-09	0,00209883
UKB-b:3881	Fresh fruit intake	MRC-IEU	M & F	446462	MRC-IEU	G	C	-0,0088917	8E-09	0,0015413
UKB-b:4080	Ankle spacing width	MRC-IEU	M & F	265753	MRC-IEU	G	C	0,0159358	2,4E-08	0,0028546

ESM Table 11. Associations of rs1800437 and phenotypes in GWAS Atlas

atlas ID	PMID	Year	Domain	Trait	P-value	N	EA	NEA
143	25673413	2015	Metabolic	Body Mass Index	1.726e-17	234069	G	C
144	25673413	2015	Metabolic	Body Mass Index (male)	1.418e-11	104666	G	C
145	25673413	2015	Metabolic	Body Mass Index (female)	4.34e-10	132115	G	C
146	25673413	2015	Metabolic	Body Mass Index	3.038e-18	236231	G	C
167	25673412	2015	Metabolic	Waist circumference	8.9e-14	232101	G	C
168	25673412	2015	Metabolic	Waist circumference	1.3e-13	244441	G	C
169	25673412	2015	Metabolic	Waist circumference (male)	1.8e-8	104406	G	C
170	25673412	2015	Metabolic	Waist circumference (male)	3.6e-8	110004	G	C
179	25673412	2015	Metabolic	Waist-hip ratio	1.6e-8	212248	G	C
180	25673412	2015	Metabolic	Waist-hip ratio	4.5e-8	226643	G	C
3185	This study	2017	Metabolic	Waist circumference	1.927e-26	385932	G	C
3186	This study	2017	Metabolic	Hip circumference	6.187e-22	385887	G	C
3270	This study	2017	Metabolic	Comparative body size at age 10	5.385e-10	379749	G	C
3435	This study	2017	Metabolic	Body mass index	9.303e-35	385336	G	C
3436	This study	2017	Metabolic	Weight	7.841e-30	385473	G	C
3440	This study	2017	Metabolic	Impedance measures - Weight	1.37e-27	379840	G	C
3441	This study	2017	Metabolic	Impedance measures - Body fat percentage	3.634e-25	379615	G	C
3442	This study	2017	Metabolic	Impedance measures - Whole body fat mass	3.128e-31	379203	G	C
3443	This study	2017	Metabolic	Impedance measures - Whole body fat-free mass	2.299e-13	379804	G	C
3444	This study	2017	Metabolic	Impedance measures - Whole body water mass	1.149e-13	379835	G	C

3445	This study	2017	Metabolic	Impedance measures - Body mass index (BMI)	2.965e-32	379831	G	C
3446	This study	2017	Metabolic	Impedance measures - Basal metabolic rate	2.072e-17	379821	G	C
3447	This study	2017	Metabolic	Impedance measures - Impedance of whole body	2.048e-10	379792	C	G
3450	This study	2017	Metabolic	Impedance measures - Impedance of arm (right)	5.559e-11	379786	C	G
3451	This study	2017	Metabolic	Impedance measures - Impedance of arm (left)	3.951e-11	379803	C	G
3452	This study	2017	Metabolic	Impedance measures - Leg fat percentage (right)	2.055e-26	379806	G	C
3453	This study	2017	Metabolic	Impedance measures - Leg fat mass (right)	1.526e-30	379802	G	C
3454	This study	2017	Metabolic	Impedance measures - Leg fat-free mass (right)	1.743e-14	379793	G	C
3455	This study	2017	Metabolic	Impedance measures - Leg predicted mass (right)	1.384e-14	379793	G	C
3456	This study	2017	Metabolic	Impedance measures - Leg fat percentage (left)	2.28e-27	379786	G	C
3457	This study	2017	Metabolic	Impedance measures - Leg fat mass (left)	2.897e-31	379783	G	C
3458	This study	2017	Metabolic	Impedance measures - Leg fat-free mass (left)	5.428e-16	379766	G	C
3459	This study	2017	Metabolic	Impedance measures - Leg predicted mass (left)	7.337e-16	379761	G	C
3460	This study	2017	Metabolic	Impedance measures - Arm fat percentage (right)	4.354e-22	379752	G	C
3461	This study	2017	Metabolic	Impedance measures - Arm fat mass (right)	1.225e-29	379725	G	C
3462	This study	2017	Metabolic	Impedance measures - Arm fat-free mass (right)	2.296e-16	379723	G	C
3463	This study	2017	Metabolic	Impedance measures - Arm predicted mass (right)	2.797e-16	379716	G	C
3464	This study	2017	Metabolic	Impedance measures - Arm fat percentage (left)	1.583e-22	379699	G	C
3465	This study	2017	Metabolic	Impedance measures - Arm fat mass (left)	6.143e-29	379663	G	C
3466	This study	2017	Metabolic	Impedance measures - Arm fat-free mass (left)	3.914e-17	379653	G	C

3467	This study	2017	Metabolic	Impedance measures - Arm predicted mass (left)	1.374e-17	379638	G	C
3468	This study	2017	Metabolic	Impedance measures - Trunk fat percentage	1.626e-20	379600	G	C
3469	This study	2017	Metabolic	Impedance measures - Trunk fat mass	1.64e-27	379578	G	C
3470	This study	2017	Metabolic	Impedance measures - Trunk fat-free mass	3.972e-10	379507	G	C
3471	This study	2017	Metabolic	Impedance measures - Trunk predicted mass	5.611e-10	379469	G	C
3939	28892062	2017	Metabolic	Body Mass Index	3.98e-9	158284	C	G
4044	30124842	2018	Metabolic	Body Mass Index	2.4e-43	681275	G	C
4074	30239722	2018	Metabolic	Body Mass Index	3.321e-59	806834	G	C
4075	30239722	2018	Metabolic	Body Mass Index (male)	1.731e-33	374756	G	C
4076	30239722	2018	Metabolic	Body Mass Index (female)	2.347e-30	434794	G	C
4077	30239722	2018	Metabolic	Waist-hip ratio	6.225e-22	697734	G	C
4078	30239722	2018	Metabolic	Waist-hip ratio (male)	3.779e-15	316772	G	C
4079	30239722	2018	Metabolic	Waist-hip ratio (female)	5.09e-10	381152	G	C
4086	30297969	2018	Endocrine	Type 2 Diabetes (adjusted for BMI)	2.7e-14	898130	C	G
4108	29403010	2018	Metabolic	Blood urea nitrogen	1.838e-10	139818	G	C

ESM Fig. 1. Schematic description of the population included in PPP-Botnia study

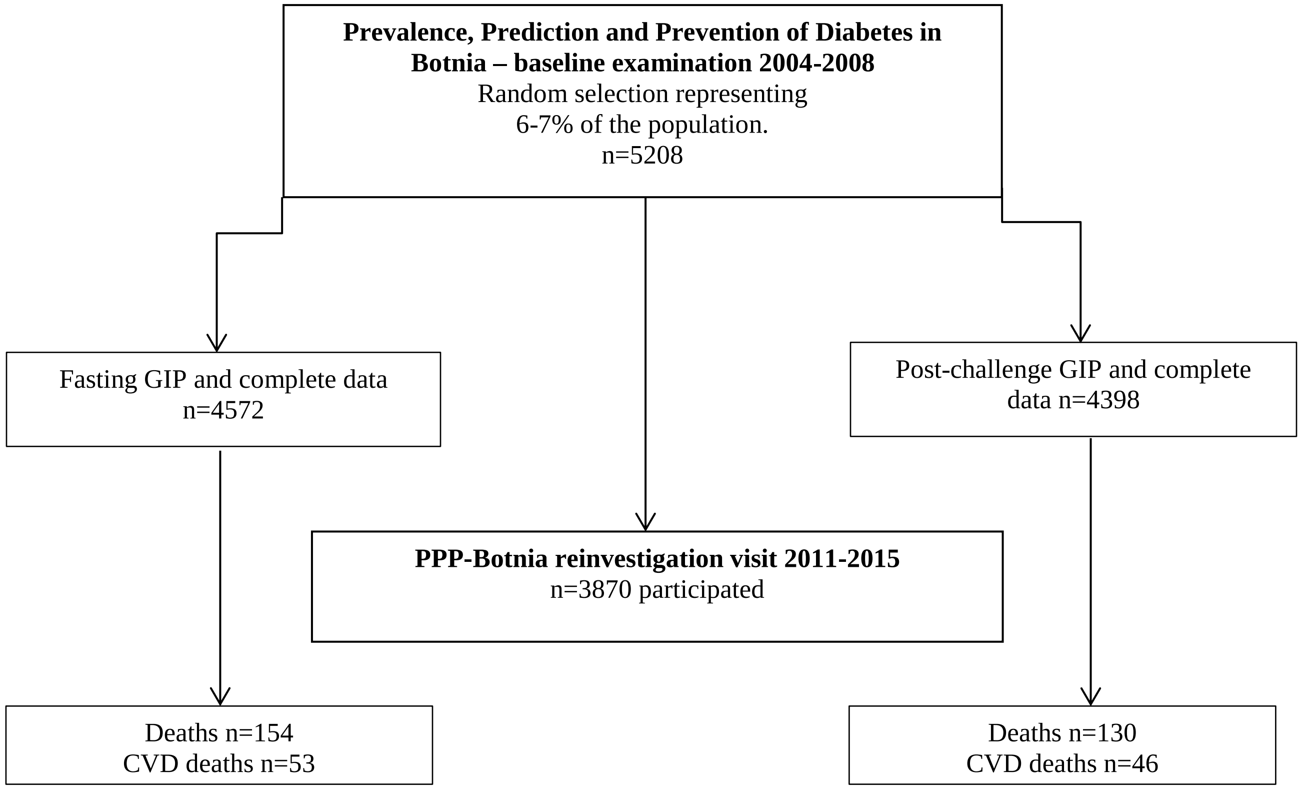

ESM Fig. 2. Schematic description of the population included in Malmö Diet and Cancer Study

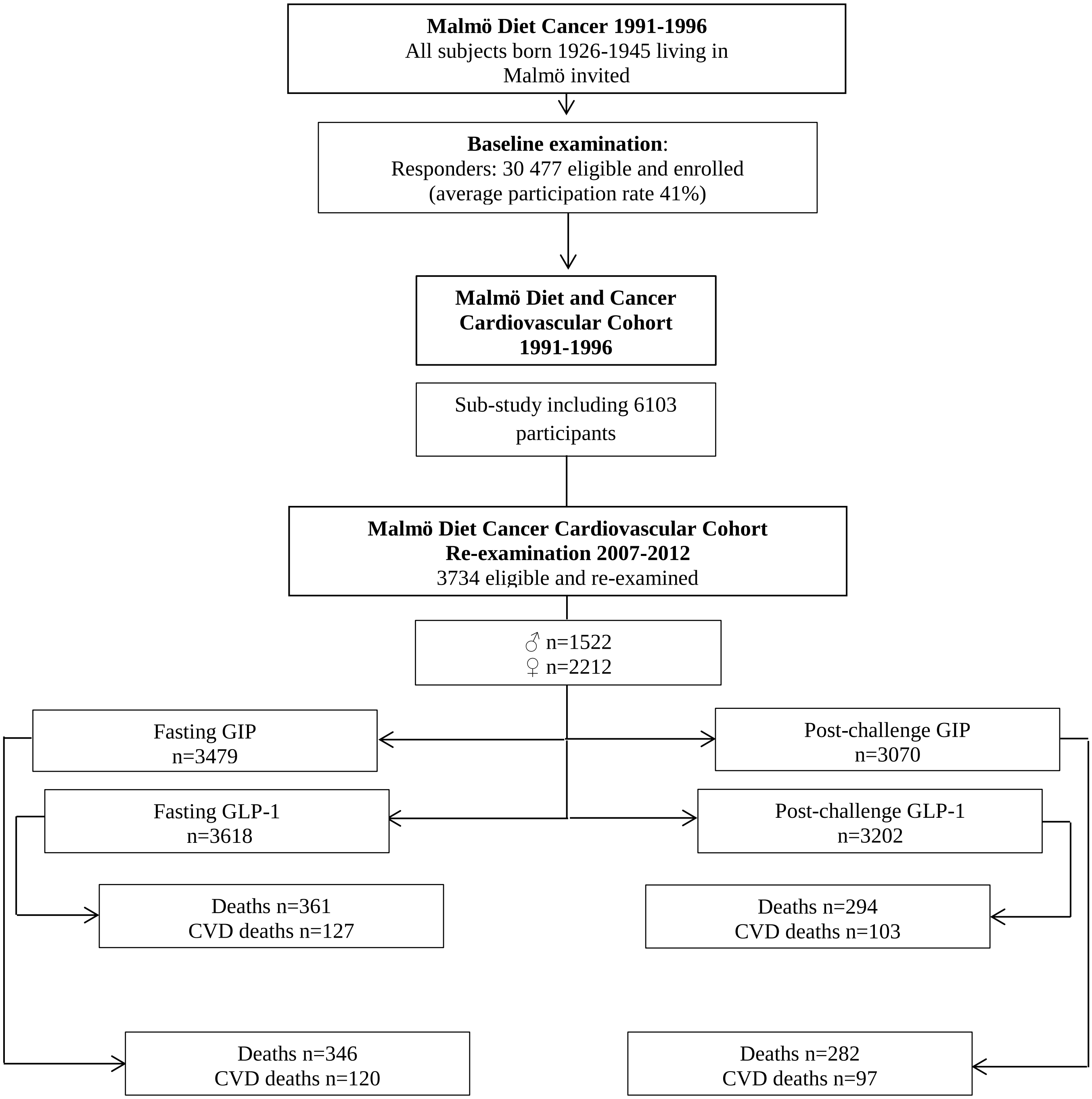

Supplement: Supplementary file 2 — (PDF 578 kb) [file 125_2020_5093_MOESM2_ESM.pdf]
